# Identification, cloning and functional characterization of novel beta-defensins in the rat (*Rattus norvegicus*)

**DOI:** 10.1186/1477-7827-4-7

**Published:** 2006-02-04

**Authors:** Suresh Yenugu, Vishnu Chintalgattu, Christopher J Wingard, Yashwanth Radhakrishnan, Frank S French, Susan H Hall

**Affiliations:** 1 Laboratories for Reproductive Biology, Department of Pediatrics, University of North Carolina, Chapel Hill, North Carolina 27599, USA; 2 Department of Physiology, Brody School of Medicine, East Carolina University, Greenville, North Carolina 27834, USA; 3 Department of Biochemistry and Molecular Biology, Pondicherry University, Pondicherry, 605014, India

## Abstract

**Background:**

beta-defensins are small cationic peptides that exhibit broad spectrum antimicrobial properties. The majority of beta-defensins identified in humans are predominantly expressed in the male reproductive tract and have roles in non-immunological processes such as sperm maturation and capacitation. Characterization of novel defensins in the male reproductive tract can lead to increased understanding of their dual roles in immunity and sperm maturation.

**Methods:**

In silico rat genomic analyses were used to identify novel beta-defensins related to human defensins 118–123. RNAs isolated from male reproductive tract tissues of rat were reverse transcribed and PCR amplified using gene specific primers for defensins. PCR products were sequenced to confirm their identity. RT-PCR analysis was performed to analyze the tissue distribution, developmental expression and androgen regulation of these defensins. Recombinant defensins were tested against E. coli in a colony forming unit assay to analyze their antimicrobial activities.

**Results:**

Novel beta-defensins, Defb21, Defb24, Defb27, Defb30 and Defb36 were identified in the rat male reproductive tract. Defb30 and Defb36 were the most restricted in expression, whereas the others were expressed in a variety of tissues including the female reproductive tract. Early onset of defensin expression was observed in the epididymides of 10–60 day old rats. Defb21-Defb36 expression in castrated rats was down regulated and maintained at normal levels in testosterone supplemented animals. DEFB24 and DEFB30 proteins showed potent dose and time dependent antibacterial activity.

**Conclusion:**

Rat Defb21, Defb24, Defb27, Defb30 and Defb36 are abundantly expressed in the male reproductive tract where they most likely protect against microbial invasion. They are developmentally regulated and androgen is required for full expression in the adult epididymis.

## Introduction

Antimicrobial proteins and peptides constitute an important part of the innate immune system of multicellular organisms including plants, insects and mammals [1]. The wide variety identified in recent years can be categorized into different structural classes including the amphipathic alpha helical peptides such as magainins in frog skin, cecropins in insects and other animals, cathelicidins in mammals and other vertebrates and the beta sheet proteins including the 2-β-strand bactenecins in mammals and the 3-β-strand defensins found in plants and animals . Among the best studied, the defensins are low molecular weight (<20 kDa) cationic peptides containing a well conserved 6 cysteine motif that forms 3 disulfide linkages. They are classified as α-, β-, and θ-defensins depending on their disulfide bond pairing and secondary structure. The α-defensins are primarily expressed in the paneth cells and polymorphonuclear leukocytes (PMNs) whereas β-defensins are expressed primarily in the epithelial cells [2]. β-defensins exhibit remarkable antibacterial, antifungal and antiviral activities against a wide variety of microorganisms [3]. Their mechanisms of action are well documented and involve the permeabilization of target cell membranes and interference with the basic metabolic processes [3].

Infections of the male reproductive tract are common and pose a threat to fertility. Epididymitis can lead to epididymal tubule damage and occlusion of the ductules by peritubular fibrosis resulting in transient or permanent sterility [4]. The innate immune responses in the male reproductive tract to microbial attack are poorly understood in spite of their likely importance in preventing the establishment and spread of sexually transmitted diseases. Microbial protection of the male tract represents such a crucial function for individual and species survival that it alone may have driven the evolution of more than 30 beta-defensin genes in 5 separate chromosomal regions [5]. This protective role is suggested by *in vivo *and *in vitro *demonstrations of their antimicrobial action. [6-12], However, β-defensins in the male reproductive tract [13-16] are also recognized key effector molecules in reproductive processes of sperm maturation and capacitation [17-19]. In addition, β-defensins exhibit chemokine properties [20]. They opsonize bacteria, inhibit the production of cortisol, act as inhibitors of protein kinase C [2] and thus may serve as an important bridge between the innate and adaptive immune systems.

In the rat, several recent studies conducted to characterize and analyze the expression of novel defensins were focused on rat genes orthologous to those on human chromosome 8p [21-25]. In a comprehensive mammalian evolutionary study, Patil *et al*. [16] reported genitourinary tract expression of 37 rat defensin genes in four clusters including those in this study [22]. Here we report a more detailed analysis specifically focused on rat *Defb21, Defb24, Defb27, Defb30 *and *Defb36 *demonstrating developmental regulation for all five genes in both epididymis and testis. We also extend the observations of Patil *et al*. by analyzing androgen dependent expression and antibacterial function. Despite the demonstration in several other species of representative defensin antibacterial activity [7,26,27], direct quantitative analysis of rat β-defensin bactericidal action has not been reported. In this study, we demonstrate potent dose and time dependent antibacterial activity of DEFB24 and DEFB30.

## Materials and methods

### Genomics

Using human *DEFB118-123 *sequence, the rat genome was searched using the BLAST program at the NCBI website , to identify the rat orthologs. Intron spanning primers were designed and RT-PCR performed using rat epididymis mRNA as the template. The specific products were sequenced and deposited in GenBank. The corresponding exon/intron boundaries were determined by aligning the cDNA with the genomic sequence. The sequences were translated using the ExPASy website .

### Tissue specimens and RT-PCR

Wistar rat (60–90 day old) tissues were obtained commercially (Zivic Laboratories Inc, Pittsburgh, PA, USA). Tissues were placed in RNA*Later *(Ambion, Inc Austin TX, USA) solution overnight at 4°C to allow penetration and fixation. The tissues were shipped on dry ice. Upon arrival, tissues were immediately stored at -70°C. Each tissue was homogenized in TRIzol reagent (Invitrogen, Carlsbad, CA, USA) and total RNA was extracted from the following tissues: caput, corpus, cauda, testis, seminal vesicle, prostate, spleen, heart, lung, liver and kidney from a single adult male and ovary, uterus, mammary gland and cervix from a single adult female. Total RNA (2 μg) was reverse transcribed using 50 U Stratascript (Stratagene, La Jolla, CA, USA) and 0.5 μg of oligodT (Invitrogen) according to the manufacturer's instructions. 2 μl of the resultant cDNA was amplified by PCR using gene specific primers (Table 1). The number of cycles to amplify each cDNA in the linear range was determined by preliminary PCR under the following conditions: 94°C for 1 min followed by 25–35 cycles at 94°C for 30 sec, 58°C for 30 sec and 72°C for 30 sec, and with a final round of extension at 72°C for 10 min. *Defb21-36 *were amplified for 32 cycles and *Gapdh *for 28 cycles. PCR amplified gene products were analyzed by electrophoresis on 2 % agarose gels. Identity of major amplicons was determined by sequencing at the UNC-CH Genome Analysis Facility using ABI PRISM model 377 DNA sequencer (PE Applied Biosystems, Foster City, CA, USA). Glyceraldehyde-3-phosphate dehydrogenase (*Gapdh*) expression was used as the internal control. To study the androgen regulation of *Defb *transcripts, epididymides from sham operated, castrated and testosterone supplemented Sprague-Dawley rats (n = 5 in each group) were obtained. Testosterone supplementation was supplied by a 20 mg dihydrotestosterone pellet implanted subcutaneously immediately after castration. All the animals were sacrificed 14 days after castration. Epididymides were stabilized in RNA*Later *solution and stored at -70°C till further use. All procedures were performed in accordance with the Guiding Principles in the Care and Use of Animals established by the National Institute of Health and approved by the Institutional Committee on the use of Animals in Research and Education. For studies on the developmental regulation of defensins, epididymides from 10–60 day old Wistar rats, one rat for each age, were obtained commercially (Zivic Laboratories).

**Table 1:** Gene specific primer sequences for rat β-defensins

**Gene**	**Primer sequence**
***Defb21***	**Forward – 5' ATA CCT GGA TCT ACT GTC CTA CCT 3' Reverse – 5' TTA TGT GTC CAT CCG TGA AGT C 3'**
***Defb24***	**Forward **– **5' GTC ATC ACC TTC ACC CCG GGA 3' Reverse **– **5' CAG CTT CTC TGG AAG TCT GTG CAT 3'**
***Defb27***	**Forward **– **5' CAC GAG GAA CAC CCT GGA TTT CC 3' Reverse **– **5' TGC CTA GGT CC ACCT TCG TTT CTG 3'**
***Defb30***	**Forward **– **5' GAG TGA CTT TCC TTT CCT CAG 3' Reverse **– **5' TCA GAA TTC CCA GAG GAA CCC TGG A 3'**
***Defb36***	**Forward **– **5' TTG GGC CTT CTC CCA CCA TGA AGC 3' Reverse **– **5' TGC ATC GTC TGG GCT TCC GGC TT 3'**

### Recombinant protein production

Recombinant proteins were prepared as described earlier [8]. Open reading frames that correspond to the rat DEFB24 and DEFB30 (amino acid sequence shown in bold in Figure 3) without the signal peptide was cloned into pQE80 expression vector (Qiagen, Valencia, CA). *E. coli *(OrigamiB (DE3) pLacI^q ^was transformed with vector pQE80 containing rat *Defb21 or Defb36 *cDNA according to the supplier's instructions. Fusion protein expression was induced with 1 mM isopropyl-1-thio-β-D-galactoside for 1 h at 37°C. 1% glucose was maintained in the medium to avoid baseline expression of the protein prior to induction. Bacterial lysate incubated with nickel-nitrilotriacetic acid-agarose (Qiagen) for 1 h to allow binding of His-tagged recombinant protein to the resin, was then transferred to a column, washed and eluted according to the manufacturer's recommendations. Fractions were analyzed on 10–20% gradient polyacrylamide Tris-Tricine gels and stained with Coomassie blue G250. Fractions containing purified protein were pooled and dialyzed against 10 mM sodium phosphate buffer (pH 7.4) to remove urea. The His-tagged recombinant DEFB proteins contained the following additional amino acid residues at their N-termini (MRGSHHHHHHGS) due to the construction of the vector.

### Antibacterial assays

Colony forming unit (CFU) assays were employed to test the antibacterial activity as described earlier [8]. *E. coli *was used to test the activity since it is one of the common causative agents of epididymitis. Briefly, overnight cultures of *E. coli *XL-1 blue (Stratagene, La Jolla, CA) allowed to grow to mid-log phase (A_600 _= 0.4 – 0.5) were diluted with 10 mM sodium phosphate buffer (pH 7.4). Approximately 2 × 10^6 ^CFU/ml of bacteria were incubated at 37°C with 1–10 μM DEFB24 or DEFB30 for 0–120 min. Aliquots of the assay mixture removed at 30, 60 and 120 min after incubation were serially diluted with 10 mM sodium phosphate buffer (pH 7.4) and 100 μl of each was spread on a LB agar plate and incubated at 37°C overnight to allow full colony development. The resulting colonies were hand counted and bacterial survival expressed as CFU/ml.

## Results

**Figure 1:**
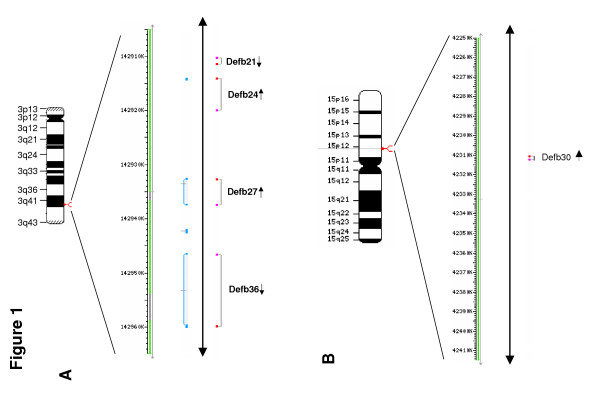
Rat *Defb *localization on Chromosomes 3 and 15. **A**, *Defb21, Defb24, Defb27 *and *Defb36 *localization rat Chromosome 3. **B**, Localization of *Defb30 *on Chromosome 15. Arrows indicate direction of transcription. Positions were taken from the MapView (build 3.1) at The National Center for Biotechnology Information (NCBI) website.

**Figure 2:**
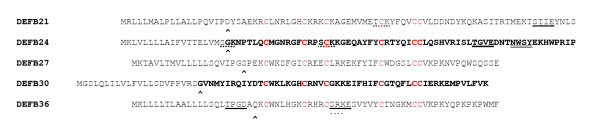
Multiple sequence alignment of rat DEFB protein sequences. Conserved 6-cysteine array is shown in red. Consensus posttranslational modification sites are indicated: double underlined – N-glycosylation; single underlined – casein kinase II phosphorylation; dotted underlined – protein kinase C phosphorylation. ^ indicates predicted signal peptide cleavage site. Sequence shown in bold was cloned to test the antibacterial activity.

Five novel β-defensin genes, *Defb21*, *Defb24*, *Defb27*, *Defb30 *and *Defb36 *were discovered in the rat genome. *Defb21*, *Defb24*, *Defb27 *and *Defb36 *are located on chromosome 3q41 and represent orthologs of the cluster on human chromosome 20q (*DEFB118*, *DEFB119*, *DEFB122 *and *DEFB123*). Orthologs of *DEFB120 *and *DEFB121 *were not found in rat. *Defb30 *on chromosome 15p12 is similar to *DEFB121*, but is orthologous to *DEFB135 *(Figure 1A and 1B). Genomic versus cDNA sequence comparisons reveal that each gene, like most β-defensin genes contains two exons (see Additional Data File 1A-C). The first exon encodes the predicted signal peptide and the second encodes a C-terminal peptide containing the characteristic β-defensin 6-cysteine motif. A PROSITE scan revealed consensus post-translational modification sites including N-glycosylation, casein kinase II phosphorylation and protein kinase C phosphorylation sites (Figure 2). Other general characteristic features of these β-defensins are listed in Table 2.

**Table 2:** General characteristic features of rat β-defensin protein isoforms.

	**DEFB21**	**DEFB24**	**DEFB27**	**DEFB30**	**DEFB36**
Length^a^(aa)	64	64	47	53	45
MW (kD)^a^	7.48	7.50	5.59	6.32	5.47
pI^a^	8.58	9.06	8.26	9.06	9.74
Cysteines^b^	6	6	6	6	6
Net Charge^a^	+3	+5	+2	+5	+10

^a ^amino acids in mature protein not including the signal peptide

^b ^number of cysteines in the C termini

**Figure 3:**
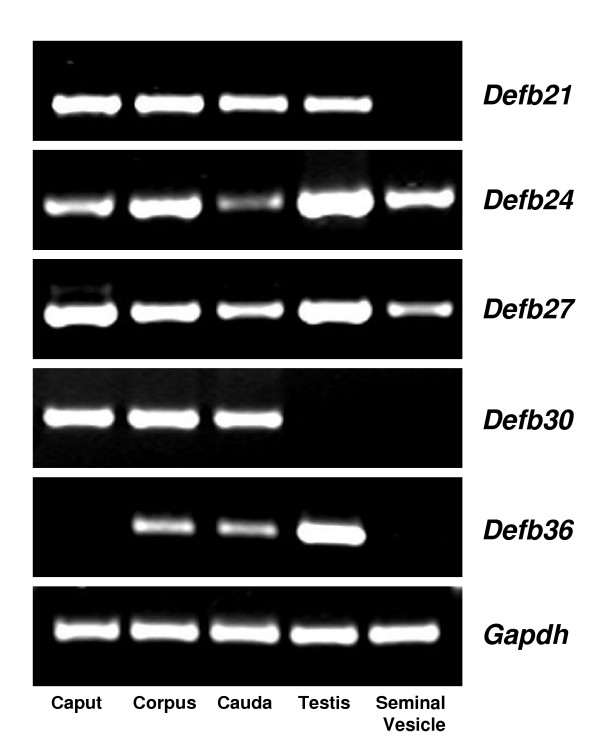
Expression of novel rat β-defensins in the male reproductive tract. Total RNAs isolated from caput, corpus, cauda, testis and seminal vesicle were reverse transcribed and PCR amplified. *Gapdh *was used as the internal control.

**Figure 4:**
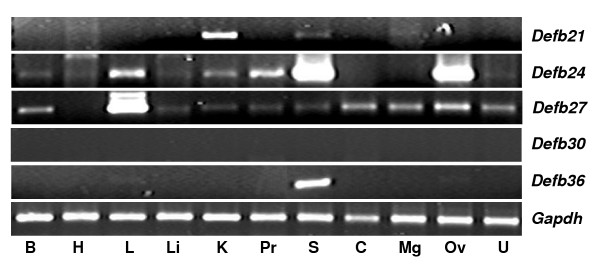
Rat *Defb *gene expression in different tissues. RT-PCR analysis was performed using total RNA isolated from **B**rain, **H**eart, **L**ung, **Li**ver, **K**idney, **P**rostate, **S**pleen, **C**ervix, **M**ammary **g**land, **Ov**ary, **U**terus. *Gapdh *was used as the internal control.

In order to understand the environment in which these *Defb *transcripts are expressed, we investigated their presence in a series of different tissues. In the male reproductive tract, *Defb21 *was expressed in all the three regions of the epididymis as well as in testis, but was not detected in the seminal vesicle (Figure 3). *Defb24 *and *Defb27 *were expressed in all male reproductive tissues analyzed (Figure 3) and many other tissues as well including the female reproductive tract (Figure 4). Expression of *Defb30 *was the most restricted, detected only in the epididymis (Figures 3 and 4). *Defb36 *expression was also restricted, detected in distal epididymis, it appeared highly expressed in testis (Figure 3) and it was also found in spleen (Figure 4).

**Figure 5:**
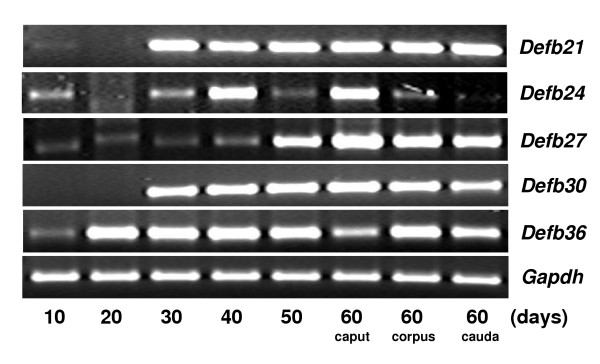
Developmental regulation of rat *Defb *genes in epididymis. RT-PCR for *Defb21, Defb24, Defb27, Defb30 *and *Defb36 *in RNA isolated from epididymides of rats aged 10–60 days.

**Figure 6:**
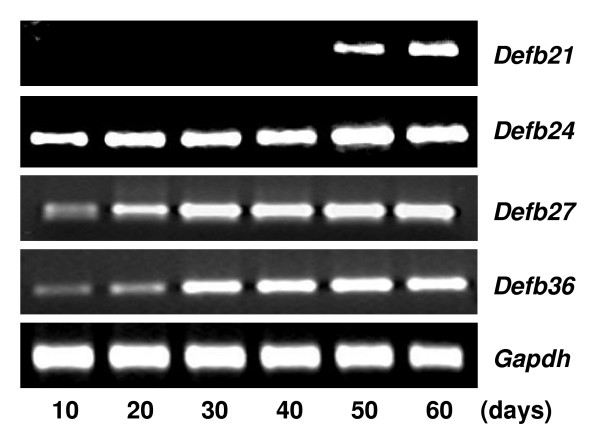
Age dependent expression of rat *Defb *in the testis. RNA from 10–60 day old rat testes were isolated and reverse transcribed followed by PCR. *Gapdh *expression served as the internal control.

**Figure 7:**
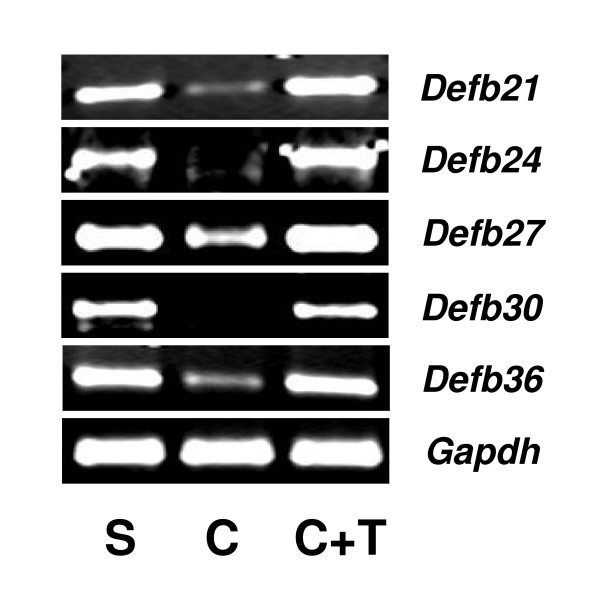
Androgen regulation of rat *Defb *genes. Rats (n = 5 for each group) were sham operated (S), castrated (C), or castrated and testosterone replaced immediately after castration (C+T). Epididymides were removed 14 days after castration. Gene expression was analyzed using RT-PCR with *Gapdh *as the internal control.

The male reproductive tract is dependent on testosterone for normal development and mature function [28]. To investigate whether androgen regulates expression of *Defb *transcripts identified in this study, age-dependent expression was analyzed in 10 to 60 day old rats. Epididymis expression of the β-defensins was variable during early development. *Defb27 *was not expressed fully until 40–50 days of age while *Defb21 *and *Defb30 *reached full expression between 20–30 days of age and *Defb36 *between 10–20 days (Figure 5). In testis, however, *Defb21 *expression was not observed until late puberty indicating a relationship to spermatogenesis (Figure 6). By contrast, *Defb24*, *Defb27 *and *Defb36 *were expressed in testis throughout this age range consistent with regulation by testosterone as well as other factors. To determine the role of testosterone in the adult epididymis, the effects of androgen ablation and replacement were investigated. Androgen ablation by castration resulted in down regulation of *Defb *expression (Figure 7). Testosterone supplementation maintained the expression of all. This result suggests testosterone involvement in regulating these defensin genes in the epididymis of adult rat.

**Figure 8:**
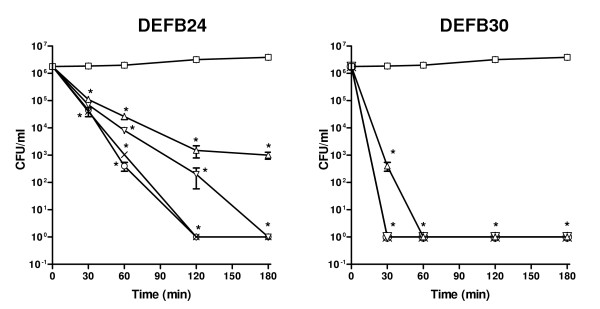
Antibacterial activity of rat DEFB24 and DEFB30. Mid-log phase *E. coli *were incubated with 1–10 μM DEFB protein for 0–120 min. (□) 0 μM; (△) 1 μM; (▽) 2 μM; (X) 5 μM; (○) 10 μM. Data (Mean ± S.E) shown are representative of three independent experiments. *p < 0.001 compared to 0 μg/ml.

Although broad spectrum antimicrobial activity of defensins and defensin-like peptides expressed in human and primate male reproductive tracts was reported previously [8,10], direct demonstration of rat β-defensin antibacterial activity has not been described. To determine if representatives of this group of rat defensins possess antibacterial capacity, their capacity to kill *E. coli *was analyzed. Both recombinant DEFB24 and DEFB30 proteins exhibited potent dose and time-dependent antibacterial activity suggesting that these rat defensins too have a role in male reproductive tract immunity (Figure 8).

## Discussion

Cationic antimicrobial peptides form an important component of innate immunity and are known to play a role in preventing the onset of infection in many organisms [29]. Systematic studies to identify and characterize novel antimicrobial proteins and peptides are revealing that the majority of defensins are expressed predominantly in the male reproductive tract [16]. Moreover, evidence is accumulating that male reproductive tract defensins not only contribute to innate immunity, but also play important roles in sperm maturation in the epididymis and capacitation [18,19,30]. What evolutionary advantage might accrue through this particular pairing of activities is not clear. It is long established and well understood that male tract functions including sperm maturation are androgen dependent [31,32]. Protection against pathogens during active sperm production years could be an inherent mechanism linked to the androgen dependent expression of these defensins and other defensin-like proteins reported previously [33-36].

Although expression of all the epididymis β-defensins exhibited some degree of androgen dependence, *Defb27 *expression appeared the least androgen dependent and yet its mRNA did not appear until 40–50 days of age in contrast to *Defb36 *that was expressed between 10–20 days. The substantial differences in age of onset of expression of the epididymis β-defensins suggest their promoters are regulated by transcription factors expressed in different developmental time frames. Further evidence indicates that this timing of expression of the epididymis β-defensins is dependent on maturation of the epithelium and consequent expression of relevant gene regulatory factors. Such factors act in concert with androgens (testosterone and dihydrotestosterone) and the androgen receptor, present in rat epididymis from the first week of postnatal life [37]. Epididymis tissue androgen decreases from birth until 20 days but remains at a substantial level of approximately 10 ng/g tissue (~35 nM) until approximately 40 days when it begins to increase to adult levels of between 15–20 ng/g [37]. The most abundant androgen in epididymis tissue during this period of postnatal development is likely dihydrotestosterone synthesized in epididymis by metabolic conversion of 5α-androstane-3α 17βdiol. During postnatal development 5α-androstane-3α 17βdiol is the major androgen produced through the actions of 5α reductase and 3α hydroxysteroid dehydrogenase and secreted by the mouse testis [38]. Testosterone is synthesized by the immature rat testis but is converted rapidly to 5α-androstane-3α 17βdiol [39-41]. Serum testosterone levels in the rat remain low and do not begin to increase to adult levels until 35–40 days [41]. Down regulation of defensin expression in the adult castrated rats in this study and the maintenance of their expression upon testosterone replacement suggests that they are regulated primarily by androgen in the adult. Androgen regulation of epididymal defensin-like gene expression was reported earlier in different species [28-33]. However, androgen-regulation of defensins outside the male tract has not been reported.

Our analyses of these five novel rat β-defensins differ on specific points from those recently reported [16]. In the male reproductive tract, we detected generally broader expression than Patil *et al*. [16]. In somatic tissues, Patil *et al*. reported *Defb36 *expression in numerous organs, whereas we found highly restricted *Defb36 *expression. The widespread expression of *Defb24 *and *Defb27 *that we report was not analyzed by Patil *et al*. but is similar to rat β-defensins *RBD-1, RBD-2 *and *Defb4 *which were also found expressed in various tissues including the male reproductive tract [16,22]. The highly restricted expression of *Defb30 *in our study is consistent with a role in protection against microorganisms specifically transmitted through the reproductive tract.

In presenting the first direct demonstration of bacterial killing by rat β-defensins we confirm the hypothesis that these proteins, related by amino acid sequence to the known antibacterial defensins of other species, indeed also possess this protective capacity. Further studies may reveal that other and perhaps all male reproductive tract defensins in rat exhibit broad spectrum antimicrobial activities and are responsible for protecting this species against pathogens *in vivo*. Male tract defensin-like bactericidal activities extend beyond *E. coli *to include *Neisseria gonorrhoeae, Staphylococcus aureus *and *Enterococcus faecalis *[11]. The antibacterial mechanisms of β-defensins in the male reproductive tract involve membrane permeabilization and inhibition of macromolecular synthesis [8-10,42]. Similar mechanisms may mediate DEFB24 and DEFB30 action. In controlling bacterial proliferation, these proteins may protect against fertility loss due to tissue damage and fibrotic occlusion of the epididymal ducts. Our studies defining the genomic, mRNA and protein sequences of these rat defensins will give impetus to further analyses to broaden our understanding of the biology of defensins, their structure-function relationships and their regulation within and beyond the male reproductive tract.

## Authors' contributions

SY performed the *in silico *analysis, PCRs, recombinant protein expression, antibacterial assays and wrote majority of the manuscript. VC and CJW conducted the androgen ablation studies. YR contributed to the genomic sequences. SHH and FSF supervised and coordinated the work and the preparation of the manuscript. All authors read, commented upon and approved the final manuscript.

## Supplementary Material

Additional File 1 Alignments of rat defensin genomic and protein sequences. Rat chromosomal sequence aligned with *Defb21*, *Defb24 ***(A)**, *Defb27*, *Defb30 ***(B) **and *Defb36 ***(C) **amino acid sequences. Exons are in upper case letters, introns in lower case. Amino acids are indicated in single letters. Numbers in parenthesis indicate amino acids of the protein. The rat cDNA sequences are available at Genbank and were assigned the accession numbers: *Defb21 *(AY600147), *Defb24 *(AY600148), *Defb27 *(AY600149), *Defb30 *(AY600146) and *Defb36 *(AY615297). Click here for file
