# Supplementary material for: Identification, cloning and functional characterization of novel beta-defensins in the rat (Rattus norvegicus)
Source: Reprod Biol Endocrinol. 2006 Feb 4;4:7. doi: 10.1186/1477-7827-4-7 (PMC1420305; doi:10.1186/1477-7827-4-7)
Supplement: Additional File 1 — Alignments of rat defensin genomic and protein sequences. Rat chromosomal sequence aligned with Defb21, Defb24 (A), Defb27, Defb30 (B) and Defb36 (C) amino acid sequences. Exons are in upper case letters, introns in lower case. Amino acids are indicated in single letters. Numbers in parenthesis indicate amino acids of the protein. The rat cDNA sequences are available at Genbank and were assigned the accession numbers: Defb21 (AY600147), Defb24 (AY600148), Defb27 (AY600149), Defb30 (AY600146) and Defb36 (AY615297). [file 1477-7827-4-7-S1.doc]

***Defb21***

aaggccgataaaactcttggccagattcatactgtacctgcgttccggatacctggatct 60

actgtcctacctcccacgaaccATGAGGCTTCTGCTGATGGCTCTTCCTCTGCTTGCTCT 120

Exon 1 M R L L L M A L P L L A L (13)

TCTACCCCAAGTGATCCCAggtaacagaaccatgggaaattaccaaaatgaaggtccggt 180

L P Q V I P (6)

aggccctggactgtgtggctcctctccctaacacaccagcaccatttttctcactgagac 240

//

ccaggctcatagccattttatagcagtaacttaaaatcgttggtctctcctttggttatt 1200

taGACTATAGTGCTGAGAAAAGATGCTTGAATAGATTGGGGCACTGCAAGAGAAAATGCA 1260

D Y S A E K R C L N R L G H C K R K C K (20)

AAGCCGGGGAAATGGTCATGGAAACATGCAAATATTTCCAAGTCTGCTGCGTTCTGGACG 1320

A G E M V M E T C K Y F Q V C C V L D D (20)

Exon 2

ACAACGACTACAAACAAAAGGCTTCCATCACGCGGACCATGGAGAAAACCTCTACAATAG 1380

N D Y K Q K A S I T R T M E K T S T I E (20)

AGTACAATCTGTCCTAAgacttcacggatggacttgacataatatagttgatggaaagat 1440

Y N L S * (4)

aattaataaataaataaataaataaataaataaataaataaaaacctgagaggtgggtag 1500

agagccctgtccctgaggtttatgatagttcctgactcatcagatgtcactcgctcctgt 1560

***Defb24***

tgtccttcagtctgctggctgaccttgtacaaatcccccctgtcccaagtcaccacccac 60

agccctgctgataagaggaggctcatccctacctcagcgaatgctgctcctattccctgc 120

agcccagtcatcaccttcaccccgggagctgccgccATGAAGCTGGTTCTCCTGCTTCTT 180

M K L V L L L L (8)

Exon 1

GCCATATTTGTGACAACGGAACTGGTAATGTCAggtaatatgggttctttacgggcctgg 240

A I F V T T E L V M S (11)

ggtcctgtccctgtatttttccatagtatagaatagagtttatttagggcatggggagga 300

//

tctttccctcctctctctttcccctctccctctcctccttcctccctccctccctctctc 5940

tttctctctctctcttggtcaGGCAAAAATCCTACCCTTCAATGCATGGGCAACAGAGGA 6000

G K N P T L Q C M G N R G (13)

TTCTGTAGGCCTTCCTGCAAAAAGGGTGAACAGGCCTACTTCTACTGCAGAACTTACCAG 6060

F C R P S C K K G E Q A Y F Y C R T Y Q (20)

Exon 2

ATATGCTGCCTCCAGTCCCATGTGAGGATCAGCCTGACAGGCGTAGAGGACAACACTAAC 6120

I C C L Q S H V R I S L T G V E D N T N (20)

TGGTCTTATGAGAAACACTGGCCAAGAATACCGTGAatgctggtacgccatgcacagact 6180

W S Y E K H W P R I P * (11)

tccagagaagctggccctgcagcctacctccttattaaaatgtatgcatctgacaatgtg 6240

***Defb27***

gtccctgctgattacaaggaacaaggatccagagctcttgatctctgccttgtgccaaca 60

ccctggatttcccgaagccATGAAGACTGCAGTATTAACTATGGTCCTGCTGCTGCTGCT 120

M K T A V L T M V L L L L L (14)

Exon 1

GTCCCAGGTCATTCCAGGTaacctaaaccccttcagaggagggacagagttggagaatag 180

S Q V I P G (6)

//

gttctggcgtagggAGCCCTGAAAAATGCTGGAAGTCTTTTGGTATCTGCCGTGAGGAGT 4800

S P E K C W K S F G I C R E E C (16)

GCCTCAGGAAAGAGAAGTTCTACATCTTCTGCTGGGATGGCAGCCTGTGCTGTGTGAAGC 4860

L R K E K F Y I F C W D G S L C C V K P (20)

Exon 2

CTAAGAATGTGCCCCAGTGGTCACAGAGTTCGGAGTAGCACTCTAGATCCCAAGGCCACA 4920

K N V P Q W S Q S S E * (11)

gaaacgaaggtggacctaggcagaggtctgagctcggcagtttctggagcttcattaaag 4980

cagctatggctggcttctgtgttcgcctctttgtatacagagtcaaatgtcagcaaaagg 4520

***Defb30***

agatgtccttgtccccaaaggttcactagccatgtacgtccttatatctagaaagctaca 60

cagagagtgactttcctttgcacctcagatgactctttgctcgctggtttgactATGGGG 120

M G (2)

Exon 1

AGCCTACAGTTGATCCTTGTGCTCTTTGTCTTGCTCTCCGATGTTCCTCCAggtaaaatg 180

S L Q L I L V L F V L L S D V P P (17)

aattcctttccttatggaattgggctatgaggttctgtaagacgaagacgaaggtggtgc 240

//

tttgattatgtagagtttcaacttaaaatgtatgaggtgccggaagtctaacttatcctt 1680

ggccatgtgacactggaggacaataactatatattaacttttccccctctgtttgcaGTT 1740

V (1)

AGAAGTGGAGTGAACATGTACATAAGACAGATTTACGATACATGCTGGAAACTAAAAGGG 1800

R S G V N M Y I R Q I Y D T C W K L K G (20)

Exon 2

CATTGCAGGAATGTATGTGGAAAAAAAGAAATTTTTCACATTTTTTGTGGTACTCAATTT 1860

H C R N V C G K K E I F H I F C G T Q F (20)

CTGTGCTGTATAGAAAGAAAGGAAATGCCCGTTCTCTTCGTGAAGTAGctgtgagtatcc 1920

L C C I E R K E M P V L F V K * (15)

agggttcctctgggaattctgagacagtcagaatggacactgcaccctgtgtcctcttga 1980

ttcctttgtccccagagtggagccacagggctggtgaacacgcagcaagcaagtttgctg 2040

***Defb36***

cgccactaactttgctctggacttgaagcttcgaagcttcgccttgggccttctcccacc 60

ATGAAGCTCCTGCTGCTGACTTTGGCTGCGCTACTACTCTTGTCCCAGCTCACTCCAGGT 120

Exon 1 M K L L L L T L A A L L L L S Q L T P G (20)

aatgtagatctcctcagggaaggcgcctggtaggcaggtagggagaccctggccttgtca 180

//

tcctcccggccagggctgccctcttcttaccacgatctgctctgtcttctttgtatcagg 13320

tGATGCTCAGAAATGCTGGAATCTCCACGGCAAGTGCCGTCACCGATGCTCCCGGAAGGA 13380

D A Q K C W N L H G K C R H R C S R K E (20)

Exon 2

AAGCGTCTATGTCTACTGCACAAACGGGAAGATGTGCTGCGTGAAGCCCAAATACCAGCC 13420

S V Y V Y C T N G K M C C V K P K Y Q P (20)

GAAGCCGAAGCCGTGGATGTTCTAActgcccggaagccggaagcccagacgatgcagatg 13480

K P K P W M F * (7)

gccaagctgtcctaggctgaccccatggactcttgagctcagtcaataaatgtgcctgcc 13520

caacctcaatgctgcccgctcatccacactagcagtgtttagtcaggggagtcagaatta 13580
